# Epidemiologie der Pflege: Prävalenz und Inanspruchnahme sowie die gesundheitliche Versorgung von Pflegebedürftigen in Deutschland

**DOI:** 10.1007/s00103-023-03693-5

**Published:** 2023-04-27

**Authors:** Antje Schwinger, Kathrin Jürchott, Chrysanthi Tsiasioti, Sören Matzk, Susann Behrendt

**Affiliations:** Forschungsbereich Pflege, Wissenschaftliches Institut der AOK, Rosenthaler Straße 31, 10178 Berlin, Deutschland

**Keywords:** Pflegebedürftigkeit, Epidemiologie, Sekundärdaten, Gesundheitsversorgung, Langzeitpflege, Long-term care dependency, Epidemiology, Administrative data, Long-term care services, Germany

## Abstract

Rund 6 von 100 gesetzlich Versicherten gelten gemäß amtlicher Statistik der Sozialen Pflegeversicherung (SPV) als pflegebedürftig. Pflegebedürftigkeit folgt hierbei analog zum öffentlichen Diskurs der sozialrechtlichen Definition und damit einem nachfrageinduzierten Verständnis von Pflege.

Um dem insbesondere demografisch bedingten Anstieg der Anzahl Pflegebedürftiger mit bedarfsgerechten Angebotsstrukturen zu begegnen, sind Kenntnis und Bewertung mehrerer Faktoren notwendig: die Häufigkeit der nach dem Elften Buch Sozialgesetzbuch (SGB XI) definierten Pflegebedürftigkeit und der unterschiedlichen Schweregrade sowie die Inanspruchnahme von Leistungen der Pflegeversicherung und der gesundheitlichen Versorgung.

Der Beitrag stellt insofern Erkenntnisse und Berechnungen aus aktuell verfügbaren administrativen Sekundärdaten der Gesetzlichen Krankenversicherung (GKV) und SPV in Deutschland vor und benennt die Limitationen dieser Daten. Im Sinne einer epidemiologischen Näherung an das Thema Versorgung Pflegebedürftiger ist eine breitere Berichterstattung auf Basis von Primärerhebungen anzustreben.

**Zusatzmaterial online:**

Zusätzliche Informationen sind in der Online-Version dieses Artikels (10.1007/s00103-023-03693-5) enthalten.

## Abstract (en)

According to official statistics from the German long-term care insurance funds, around six out of every one hundred people with statutory health insurance are considered to be in need of long-term care. In this context, “need of long-term care” is defined according to the Eleventh Book of the Social Code (SGB XI) and therefore follows a demand-driven understanding of care, which also aligns with public discourse.

In order to meet the increasing number of people in need of long term care – mostly caused by demographic change – with needs-based service structures, knowledge and evaluation of several factors are necessary: the prevalence of care dependency as defined by the SGB XI, the different degrees of severity, and the utilisation of long-term care and healthcare services.

In this respect, the article presents findings and calculations based on currently available administrative data from German health and long-term care insurance funds and states its limitations. In terms of an actual epidemiological approach to the topic of long-term care, the aim should be broader reporting based on primary surveys.

## Einleitung

Der demografische Wandel stellt uns vor enorme gesellschaftliche Herausforderungen. Bis 2050 wird in bestimmten Regionen Deutschlands mehr als jeder 10. auf pflegerische und betreuende Unterstützung angewiesen sein [1–3]. Pflegebedürftige Menschen sind in unterschiedlichem Ausmaß in ihrer Gesundheit und damit zumeist in der autonomen Lebensführung, der Mobilität und der sozialen Teilhabe beeinträchtigt. Pflegebedürftigkeit im Alter bedeutet in der Regel, dass bei den Betroffenen mehrere somatische und oftmals auch psychische Erkrankungen vorliegen; die Prävalenz von Demenzen ist hier hoch: Im Pflegeheim gelten 69 % der Bewohnenden als demenziell erkrankt [4].

4 von 5 Pflegebedürftigen werden zu Hause gepflegt und betreut. Damit findet Langzeitpflege maßgeblich in den Familien statt. Die hieraus resultierenden physischen und psychosozialen Belastungen der Angehörigen sind in vielfacher Weise untersucht [5–7]. Wissen über Pflegewahrscheinlichkeiten und -ursachen wie auch über eine effektive, präferenzgerechte pflegerische und gesundheitliche Versorgung ist heute wie zukünftig Voraussetzung, um bedarfsgerechte Angebotsstrukturen zu entwickeln, zu planen und zu steuern.

Im vorliegenden Beitrag betrachten wir die Pflege in Deutschland unter den Gesichtspunkten Prävalenz der Pflegebedürftigkeit und Inanspruchnahme von Leistungen der Pflegeversicherung sowie die gesundheitliche Versorgung von Pflegebedürftigen. Wichtig ist: Pflegebedürftigkeit folgt hier – analog zum öffentlichen Diskurs – der sozialrechtlichen Definition und damit einem nachfrageinduzierten Verständnis von Pflege. Seit nun fast 30 Jahren ist Pflegebedürftigkeit in Deutschland durch das Elfte Buch Sozialgesetzbuch (SGB XI) normiert. So zog die Einführung der Pflegeversicherung im Jahr 1995 nach sich, dass die zu einem Leistungsanspruch führenden Tatbestände festzulegen waren. Seit der Reformierung des Pflegebedürftigkeitsbegriffs im Jahr 2017 im Zuge des Zweiten Pflegestärkungsgesetzes (PSG II) liegt der Schwerpunkt auf einem ressourcenorientierten Verständnis von Pflege. Während vor 2017 die somatischen Einschränkungen im Fokus standen, sind nun u. a. auch Beeinträchtigungen von Fähigkeiten in den Bereichen „kognitive und kommunikative Fähigkeiten“, „Verhaltensweisen und psychische Problemlagen“, „Gestaltung des Alltagslebens und sozialer Kontakte“ berücksichtigt. Die sozialrechtliche Normierung ist gesellschaftlich prägend für unser Verständnis von Pflege [8] und auch die hier präsentierten Daten sind dementsprechend zu interpretieren.

Es werden zunächst die Prävalenz von Pflege sowie zu erwartende Entwicklungen und nachfolgend die Inanspruchnahme von pflegerischen Leistungen dargestellt. Beim anschließenden Blick auf die Inanspruchnahme medizinischer Leistungen werden die verfügbaren Daten zu Unter- und Fehlversorgung von Pflegebedürftigen ausgewertet und diskutiert. Abschließend werden die Relevanz und Verfügbarkeit öffentlicher Berichterstattung und amtlicher Statistiken zur Langzeitpflege zusammengefasst. Die präsentierten Ergebnisse basieren auf der Amtlichen Statistik PG 2 „Leistungsempfänger nach Pflegegraden, Altersgruppen und Geschlecht“ des Bundesministeriums für Gesundheit. Darüber hinaus werden verschlüsselte Abrechnungsdaten der AOK sowie – für deren Standardisierung – die Amtliche Statistik über die Versicherten der Gesetzlichen Krankenversicherung (GKV; KM 6) verwendet.

## Pflegeprävalenzen und Inanspruchnahme von Pflegeleistungen

### Prävalenz der Pflegebedürftigkeit

Rund 4,6 Mio. gesetzlich Versicherte galten Ende des Jahres 2021 laut amtlicher Statistik der Sozialen Pflegeversicherung (SPV) als pflegebedürftig, knapp 2 Drittel (61,6 %) hiervon sind Frauen (Abbildung Z1 im Onlinematerial). Die Hälfte der Pflegebedürftigen (52,1 %) sind 80 Jahre und älter. Gleichwohl zählen zu den Pflegebedürftigen auch rund 270.000 Kinder und Jugendliche (< 20 Jahre; 5,9 %) sowie rund 600.000 Menschen im erwerbsfähigen Alter (20–59 Jahre; 13,2 %). Mit dem Alter steigt die Wahrscheinlichkeit für eine Pflegebedürftigkeit – von 4 % bei den 60- bis 64-Jährigen auf 10 % bei den 70- bis 74-Jährigen und 70 % bei den über 90-Jährigen (Abbildung Z2 im Onlinematerial). Bis zum 80. Lebensjahr ist die Pflegewahrscheinlichkeit zwischen den Geschlechtern in etwa gleich verteilt, danach zeigen sich jedoch deutliche Unterschiede. So gelten 60 % der über 90-jährigen Männer als pflegebedürftig, bei den gleichaltrigen Frauen betrifft dies 73 % (Abbildung Z2 im Onlinematerial).

Seit Einführung des gesetzlich neuen Pflegebedürftigkeitsbegriffs im Januar 2017 unterteilt sich die Schwere der Pflegebedürftigkeit in 5 Pflegegrade (zuvor 3 Pflegestufen). Am häufigsten sind die Pflegebedürftigen dem Pflegegrad 2 („erhebliche Beeinträchtigungen“, 40,6 %) zugeordnet, gefolgt von Pflegegrad 3 („schwere Beeinträchtigungen“, 28,2 %; siehe Abbildung Z3 im Onlinematerial). Der durchschnittliche Pflegegrad hat sich dabei seit 2017 deutlich verändert. Der niedrigschwellige Pflegegrad 1 („geringe Beeinträchtigungen der Selbständigkeit oder der Fähigkeiten“) wurde geschaffen, um das erklärte Ziel der Reform – einen verbesserten Zugang zur Pflege – zu gewährleisten. Der Anteil der Pflegebedürftigen in dieser Kategorie hat sich seit seiner Einführung im Jahr 2017 mehr als verdoppelt (von 5,8 % im Jahr 2017 auf 13,8 % im Jahr 2021, entspricht nunmehr 1 Mio. Pflegebedürftigen). Es liegt nahe, dass dieser extreme Anstieg von Anspruchsberechtigten in Pflegegrad 1 als Einführungseffekt dieser letztlich neuen Zugangsart in die Pflege zu interpretieren ist (siehe Abbildung Z3 im Onlinematerial).

**Figure 1:**
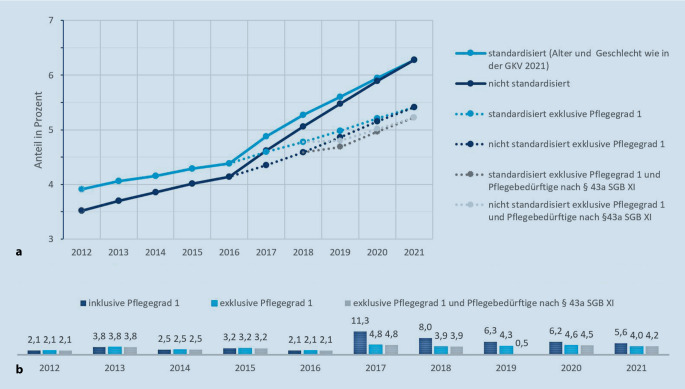

Im Jahr 2012 waren 3,5 % der gesetzlich Versicherten pflegebedürftig im Sinne des SGB XI. Ende 2021 lag dieser Anteil bei 6,3 %, was einem Anstieg von rund 80 % in den letzten 10 Jahren entspricht. Bereinigt man die Pflegeprävalenz um den Alterungseffekt der Gesellschaft und legt auch für das Jahr 2012 die gleiche Alters- und Geschlechtsstruktur wie im Jahr 2021 zugrunde, ergäbe sich eine Pflegeprävalenz von 3,9 % im Jahr 2012 und damit ein Plus von 60 % bis 2021. Auch hier liegt der Prävalenzsprung hauptsächlich in der Einführung des neuen Pflegebedürftigkeitsbegriffs im Jahr 2017 begründet (Abb. 1). Lässt man Pflegegrad 1 bei den Zeitverlaufsbetrachtungen außen vor, da es vor 2017 kein Pendant hierzu gab, verändert sich die Prävalenzentwicklung deutlich (Abb. 1). Die Pflegeprävalenz wäre – bereinigt um den Alterungseffekt der Bevölkerung – dann nur noch um 40 % gestiegen. Sichtbar wird: Auch 5 Jahre nach Einführung des Pflegebedürftigkeitsbegriffs steigt die Pflegeprävalenz deutlich über das demografisch zu erwartende Niveau. Hieraus ergeben sich wichtige Forschungsfragen zu möglichen Prädiktoren, wie etwa epidemiologischen (z. B. Anstieg von Demenzen), angebots- und nachfrageinduzierten (z. B. bessere Verfügbarkeit und Bekanntheit der Angebote), sozioökonomischen (z. B. Zunahme von Single-Haushalten, Absinken des verfügbaren Haushaltseinkommens/Abhängigkeit von Transferleistungen der SPV) und gesellschaftlich-normativen (z. B. positive Wertung/keine Assoziation von Stigmatisierung bei Pflegebedürftigkeit). Ebenso ist zu eruieren, ob die während der COVID-19-Pandemie neu eingeführte Begutachtung auf Basis strukturierter Telefoninterviews sowie weitere pandemiebedingte Faktoren eine Auswirkung auf die Pflegeprävalenz hatten.

In der Gesamtschau treten die Limitationen einer Pflegeberichterstattung, die auf einem sozialrechtlich normierten Verständnis von Pflege aufbaut, deutlich zu Tage. Perpetuiert werden die Ungewissheiten, wenn die im vorherigen Absatz skizzierten möglichen Einflussfaktoren in Prognosen von Pflegeprävalenz und -bedarf eingehen. Trotz dieser Limitationen wird in Studien die bis zum Jahr 2050 erwartete Pflegeprävalenz auf bis zu 8 Mio. Pflegebedürftige geschätzt (jüngere Arbeiten sind [1–3]).

### Verweildauer und Versorgungsformen bei Pflegebedürftigkeit

**Figure 2:**
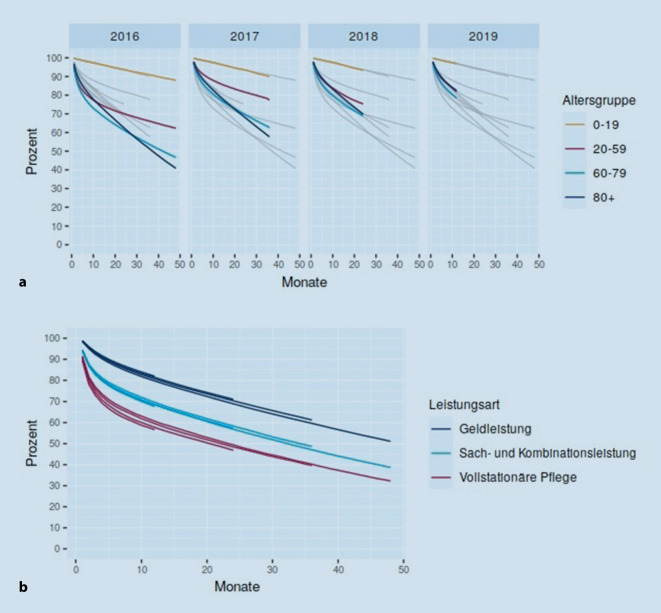

Der Einfluss der gesetzlichen Anpassung des Pflegebedürftigkeitsbegriffs und des damit verbundenen veränderten, insbesondere auch niedrigschwelligen Zugangs zur Pflege zeigt sich auch bei den Verweildauerverläufen. Abb. 2 zeigt die Bezugsdauer von Pflegeleistungen nach Alter und Versorgungsform, wobei die Verweildauerkurven sich auf das jeweilige Eintrittsjahr in die Pflege beziehen. Kinder und Jugendliche haben erwartungsgemäß einen langen Verbleib in der Pflege (nach 3 Jahren waren noch rund 90 % pflegebedürftig). Auch Pflegebedürftige im erwerbsfähigen Alter zeigen hohe Verweildauerraten. Zu Tage treten beispielsweise Unterschiede zwischen den Vor- und Nach-Pflegereform-Kohorten: Bei Pflegeeintritt im Jahr 2016 überlebten nur 65 % 36 Monate und länger, bei Eintritt ein Jahr später 76 %. Zudem verschiebt sich die Verweildauerkurve der Gruppe 80-plus deutlich. Bezogen in der 2016-Kohorte nach 3 Jahren 55 % Pflegeleistungen, waren es in der 2017-Kohorte noch 64 % (Abb. 2). Differenziert nach der zu Beginn der Pflegebedürftigkeit gewählten Leistungsart zeigt sich, dass Personen, die mit dem Einstieg in die Pflege vollstationär betreut wurden, deutlich kürzere Verläufe in der Pflege haben als jene mit reiner Geldleistungsunterstützung. So endet bei rund 60 % der Pflegeheimbewohnenden nach 36 Monaten der Leistungsbezug, bei den Pflegegeldempfangenden im Vergleich nur bei rund 40 % (Abb. 2). Hervorzuheben ist: Hier dargestellt sind ausschließlich Verweildauerraten für neu in die Pflegebedürftigkeit Eingetretene, d. h. inzidente Pflegeheimkohorten. Die Ergebnisse sind folglich nicht für die Diskussion der 2022 eingeführten Begrenzung der Eigenanteile nach Wohndauern in der vollstationären Pflege (§ 43c SGB XI) zu nutzen – hierfür bedarf es Verweildauerbetrachtungen der Gesamtkohorte der Pflegebedürftigen im Heim.

**Figure 3:**
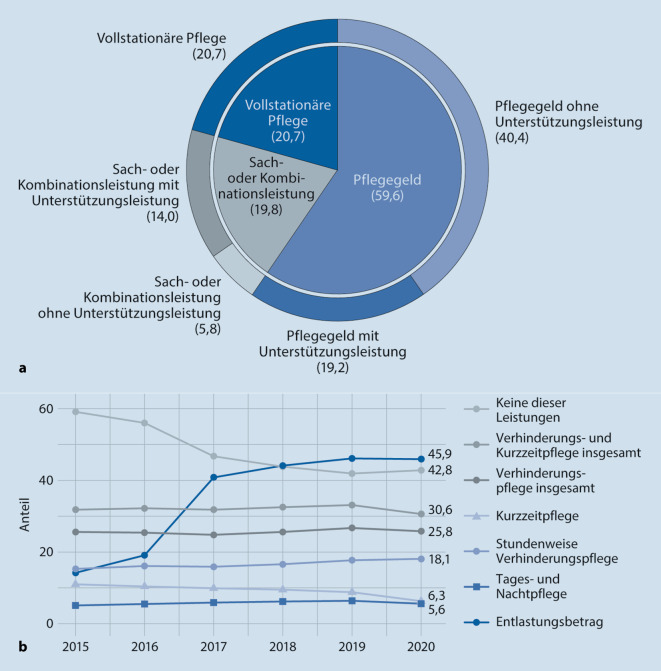

Der Anteil vollstationär versorgter Pflegebedürftiger sank ferner in den letzten Jahren relational und absolut. War 2015 jeder 4. Pflegebedürftige (26,1 %) vollstationär versorgt, so war es 2020 nur noch jeder 5. (20,7 %; [9]). Ein weiteres Fünftel (19,8 %) nutzte 2020 einen Pflegedienst (Sach- und Kombinationsleistung), 3 Fünftel (59,6 %) bezogen Pflegegeld (Abb. 3). Ambulant versorgten Pflegebedürftigen stehen zusätzlich zum Pflegegeld bzw. parallel zur ergänzenden Versorgung durch einen Pflegedienst weitere Unterstützungsleistungen zur Verfügung. Neben der Tages- und Nachtpflege (§ 41 SGB XI), der Verhinderungspflege (§ 39 SGB XI) und der Kurzzeitpflege (§ 42 SGB XI) ist dies der Entlastungsbetrag von 125 € monatlich (§ 45b SGB XI). Abb. 3 zeigt die Inanspruchnahme der verschiedenen Unterstützungsleistungen (mit und ohne Pflegedienst) sowie die zeitliche Entwicklung der Inanspruchnahme in den Jahren 2015 bis 2020. Besonders auffällig ist dabei: 40 % aller Pflegebedürftigen nutzen keine einzige weitere ambulante Unterstützungs- und Entlastungsleistung.

Die Inanspruchnahme der ambulanten Unterstützungs- und Entlastungsleistungen hat von 2015 auf 2019 zugenommen, zuvorderst die Nutzung der stundenweisen Verhinderungspflege und des Entlastungsbetrags (Abb. 3). Der Zugang zu den Unterstützungsangeboten wurde gleichwohl in einer ganzen Reformkaskade deutlich ausgeweitet. Seit 2015 kann die Tagespflege gänzlich additiv zur Sach‑, Kombinations- oder Geldleistung genutzt werden. Verhinderungs- und Kurzzeitpflege können ebenso seit 2015 anteilsmäßig substituiert werden und der vormals auf demenziell Erkrankte beschränkte Anspruch auf Betreuungsleistungen bzw. niedrigschwellige Entlastungen wurde auf alle Pflegebedürftigen ausgeweitet. Eine dezidierte Bewertung der Inanspruchnahme steht indessen aus, denn die Nachfrage ist schlussendlich präferenzgeleitet. Für Deutschland gibt es keine Leitlinien dazu, ab welchem Pflegeumfang welche Unterstützung mit Blick auf ein gewünschtes Ergebnis effektiv und damit empfohlen ist. Allein eine ganze Reihe an Befragungen beschäftigen sich mit der Wahrnehmung und Inanspruchnahme dieser Angebote sowie mit entsprechenden Barrieren bei den Verbraucherinnen und Verbrauchern [5–7, 10–13]. Die Studien verweisen ferner auf das Ausmaß der Einbindung von familiärer Pflege: Im Durchschnitt wird für zu Hause Gepflegte ein wöchentlicher Pflege- und Unterstützungsbedarf von rund 60 h und mehr erhoben [7, 10, 13]. Auch die hieraus resultierenden Belastungen treten in den Befragungen deutlich zu Tage: Jede 3. bis 4. Hauptpflegeperson kann die Pflegesituation nach eigener Auskunft „nur noch unter Schwierigkeiten“ oder „eigentlich gar nicht mehr“ bewältigen [5–7].

## Gesundheitliche Versorgung von Pflegebedürftigen

Pflegebedürftige weisen eine höhere Morbidität auf als Nicht-Pflegebedürftige, wobei Pflegeheimbewohnende nochmals stärker betroffen sind als im häuslichen Setting Gepflegte [14]. Gleichzeitig findet die Versorgung der Pflegebedürftigen an systemischen Schnittstellen statt – insbesondere an jenen der pflegerischen und ambulant-ärztlichen Versorgung sowie mit Blick auf die Hospitalisierung und Arzneimittelversorgung.

### Ambulant-ärztliche Versorgung

**Figure 4:**
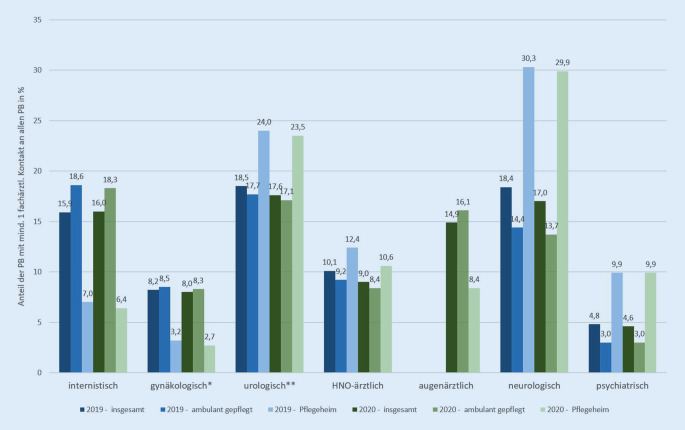

Die ambulant-ärztlichen Leistungen besitzen zusammen mit der pflegerischen Versorgung einen zentralen Stellenwert für Pflegebedürftige, denn nahezu jede pflegebedürftige Person (96 %) weist mindestens einen vertragsärztlichen Kontakt auf (Tab. 1-I). Etwas mehr als 2 Drittel der Pflegebedürftigen sind auch in fachärztlicher Behandlung. Bei der Inanspruchnahme der einzelnen fachärztlichen Gruppen zeigen sich jedoch starke Unterschiede, die auf die Bedarfs- und Morbiditätsstrukturen hinweisen. Auch wenn demenzielle Erkrankungen nicht die einzige Indikation für eine neurologische Behandlung darstellen, gibt Abb. 4 auch Aufschluss über die Demenzprävalenz bei Pflegebedürftigen. Der Anteil von Pflegeheimbewohnenden mit Inanspruchnahme neurologischer Leistungen ist mit 29,9 % doppelt so hoch wie der Anteil unter häuslich Gepflegten (13,7 %; [9]). Zum anderen gibt Tab. 1-I Hinweise auf Bereiche der fachärztlichen Unterversorgung. Mehr als ein Viertel der Pflegebedürftigen (2019: 28,2 %; 2020: 29,4 %) hat keinen fachärztlichen Kontakt. Schulz et al. formulieren in treffender Weise das gefühlte Paradoxon: Während die Morbidität von betagten Langzeitgepflegten wesentlich komplexer und umfassender ist als bei Personen ohne Langzeitpflege, ist in der erstgenannten Gruppe die morbiditätsbereinigte Wahrscheinlichkeit für einen fachärztlichen Kontakt wesentlich geringer – und zwar bei 10 von 12 in der Studie betrachteten Fachdisziplinen. Lediglich auf die Bereiche Neurologie und Psychiatrie trifft dies nicht zu [14].

**Table 1:** 

***I – Ambulant-ärztliche Versorgung***^*a,b,c*^				
	*PB mit Fall in Relation zu allen PB [%]*		*Fälle von PB in Relation zu allen Fällen von PB* *+* *NPB [%]*	
	**2019**	**2020**	**2019**	**2020**
Gesamt – vertragsärztlicher Kontakt	95,9	95,7	–	–
Hausärztlich/hausärztlich-internistisch	89,7	88,6	10,6	12,2
Fachärztlich	71,8	70,6	8,9	10,1
***II – Kritische Arzneimitteleinsätze***^*a,b*^				
	*PB mit Verordnung in Relation zu allen PB [%]*		*NPB mit Verordnung in Relation zu allen NPB [%]*	
Polymedikation (5+ Wirkstoffe in jedem Quartal des Jahres)	61,2	60,3	11,6	10,7
Davon:				
5 bis 6	20,0	19,8	6,4	5,9
7 bis 9	23,4	23,1	3,8	3,5
10+	17,8	17,4	1,4	1,3
Mind. 1 Verordnung eines Wirkstoffs der PRISCUS-Liste	14,5	13,9	7,9	7,5
Mind. 1 Antipsychotikaverordnung	17,2	16,0	1,5	1,5
***III – Hospitalisierung***^*a*^				
	*PB mit Hospitalisierung in Relation zu allen PB [%]*		*NPB mit Hospitalisierung in Relation zu allen NPB [%]*	
Mind. eine Hospitalisierung ^b^	18,4	16,0	–	–
	*PB*		*NPB*	
Zahl der Hospitalisierungen je Person [n] ^d^	2,1	2,0	1,5	1,4
Anteil Hospitalisierungen an allen Hospitalisierungen ^b^	26,0	29,1	74,0	70,9
Tage je Hospitalisierung [n] ^d^	8,2	8,3	5,1	4,9
Anteil Tage an allen Krankenhaustagen [%] ^b^	39,2	43,5	60,8	56,5

*NPB* Nicht-Pflegebedürftige. Berücksichtigt werden nur PB und NPB im Alter von 65+ Jahren

^a^ Alle Daten basieren auf AOK-Daten und sind standardisiert auf die gesetzlich Versicherten (Amtliche Statistik KM 6 2020 bzw. 2019)

^b^ Die Ergebnisse stellen den Durchschnitt der Quartale des jeweiligen Kalenderjahres dar

^c^ Fälle aus Selektivverträgen nach § 73 oder § 140a Fünftes Buch Sozialgesetzbuch (SGB V) sind hier exkludiert

^d^ Ergebnisse beziehen sich auf das Kalenderjahr

*Quelle*: eigene Darstellung, nach Pflege-Report 2021 [29] und 2022 [9]

Am Beispiel der Diabetesversorgung ergeben sich aus Abb. 4 ebenso Indizien für eine fachärztliche Unterversorgung: Angesichts einer Prävalenz von Diabetes mellitus Typ 1 und Typ 2 von 43 % bei Pflegeheimbewohnenden [15] und der Leitlinienempfehlung einer jährlichen augenärztlichen Untersuchung bei Risikopopulationen [16, 17] erscheint der Anteil der Bewohnenden, die augenärztlichen Kontakt hatten, mit 8,4 % (2020; ambulante Pflege: 16,1 %) gering [9].

### Kritische Arzneimitteleinsätze

Ein zentraler Aspekt der Versorgung von Pflegebedürftigen ist das komplexe Feld der Arzneimittelversorgung. Die folgenden Ausführungen (mit Daten für das Jahr 2020) konzentrieren sich hierbei auf 3 potenziell kritische Arzneimitteleinsätze: Polymedikation (der gleichzeitige Einsatz mehrerer Medikamente), die für ältere Menschen risikobehafteten Wirkstoffe der PRISCUS-Liste [18] sowie die langfristige Verordnung von Antipsychotika bei Demenz. Insbesondere die in der Regel vorliegende Multimorbidität, physiologische Veränderungen und eine reduzierte Immunabwehr im Alter der Betroffenen erhöhen das Risiko für das Auftreten von unerwünschten Arzneimittelwirkungen und stellen damit zentrale Herausforderungen an eine adäquate medikamentöse Therapie dar [4]. Durch Polymedikation potenziert sich schließlich die Gefahr von wirkstoffbezogenen Wechselwirkungen bei Pflegebedürftigen. Im Jahr 2020 nahmen 60 % der über 65-Jährigen mindestens 5 Wirkstoffe in jedem Quartal des Jahres ein. Zum Vergleich: Bei Nicht-Pflegebedürftigen betrug dieser Anteil im selben Jahr 11 % (Tab. 1-II). Ferner erhielt jede 7. pflegebedürftige Person (13,9 %) eine risikobehaftete Arzneimittelverordnung der PRISCUS-Liste (Tab. 1-II). Auch weist knapp jede 6. pflegebedürftige Person (16,0 %,) eine Antipsychotikaverordnung im Durchschnitt der Quartale auf (Tab. 1-II). Bei rund 6 % der Pflegebedürftigen kommen hierbei antipsychotische Wirkstoffe zum Einsatz, die auf der PRISCUS-Liste zu finden sind [9]. Die Fehlversorgung mit Psychopharmaka und hier insbesondere der Einsatz von Antipsychotika bei Demenz ist ein auch international gut untersuchtes Versorgungsthema bei Betagten [2]. Während der klinische Nutzen als moderat eingeschätzt wird, sind die Risiken eines Antipsychotikaeinsatzes bei Demenz erheblich [4]. Nicht zuletzt angesichts der hohen Prävalenz von demenziellen Erkrankungen bei Pflegebedürftigen stellt der Umgang mit den im Verlauf einhergehenden psychischen Störungen und Verhaltensstörungen wie Apathie, Aggressivität oder auch gestörten Tag-Nacht-Rhythmen eine Herausforderung dar. Antipsychotika sind hier als letztes Mittel der Wahl und dann kurzzeitig, niedrigst dosiert und engmaschig kontrolliert einzusetzen [19].^1^

**Figure 5:**
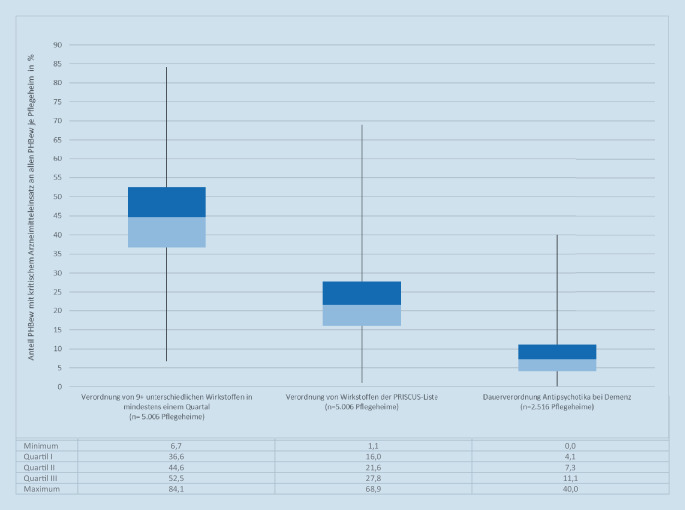

Derartige potenzielle Versorgungsdefizite sind ferner auf Ebene der Pflegeheime und der dortigen Leistungserbringenden der unterschiedlichen Berufsgruppen zu berichten. Abb. 5 zeigt die routinedatenbasierten Qualitätsindikatoren für Polymedikation (hier definiert ab 9 und mehr Wirkstoffe je Quartal), die Verordnung von Wirkstoffen der PRISCUS-Liste (Version von 2010) sowie für den Dauereinsatz von Antipsychotika bei Demenz. Diese Indikatoren entstammen dem Innovationsfonds-Projekt *QMPR – Qualitätsmessung in der Pflege mit Routinedaten*, das für die einzelne Pflegeeinrichtung die Prävalenz von Versorgungsauffälligkeiten berechnet [20]. Die Boxplots stellen die Verteilung der unadjustierten Anteile der Bewohnenden mit kritischem Arzneimitteleinsatz je Pflegeheim nach Quartilen dar. Bei der Hälfte der Pflegeheime (Quartil III und IV) sind mindestens 45 % der Bewohnenden polymedikamentös versorgt. Ebenso in der Hälfte der Pflegeheime weist mindestens ein Fünftel (21,6 %) der Bewohnenden PRISCUS-Verordnungen auf. In einem Zehntel der Einrichtungen (11,1 %) kommen bei demenziell Erkrankten dauerhaft Antipsychotika zum Einsatz (mind. 30 angenommene mittlere Tagesdosen „defined daily dose“, DDD) in 2 aufeinanderfolgenden Quartalen).

### Hospitalisierung

Krankenhausaufenthalte markieren eine von Pflegebedürftigen häufig frequentierte Schnittstelle der Versorgung: Im Jahr 2020 gingen 44 % aller Tage im Krankenhaus auf die Behandlung Pflegebedürftiger zurück (Tab. 1-III). Bei der Anzahl der Krankenhausaufenthalte gingen im Jahr 2019 etwas mehr als ein Viertel (26,0 %) und im Jahr 2020 fast ein Drittel (29,1 %) auf Pflegebedürftige zurück. Im Jahr 2020 war jede pflegebedürftige Person im Schnitt zweimal im Krankenhaus. Ein Aufenthalt dauerte dabei rund 8 Tage (Tab. 1-III). Insbesondere im Bereich der Hospitalisierung von Pflegebedürftigen lassen sich deutliche Effekte der COVID-19-Pandemie feststellen. So sank die Hospitalisierungsprävalenz (Anteil der Pflegebedürftigen mit mindestens einer Hospitalisierung) von 2019 auf 2020 zwar um „lediglich“ 2,4 Prozentpunkte auf 16 % (Tab. 1-III), für Pflegeheimbewohnende jedoch zeigte die Studie *Covid-Heim* einen Rückgang von Krankenhausaufnahmen um rund ein Drittel für den Zeitraum der ersten Pandemiewelle (10.03. bis 30.06.2020) im Vergleich zu den Vorjahren 2015 bis 2019. Ursächlich hierfür ist primär der Ausschluss nicht akuter Versorgungsleistungen in Krankenhäusern. Dass auch Einweisungen mit Notfallindikationen bei Pflegeheimbewohnenden abnahmen, lässt jedoch auch auf Unter- und Fehlversorgung im Setting Pflegeheim schließen [21].

Grundsätzlich gilt jedoch: Transfers von der Häuslichkeit oder vom Pflegeheim ins Krankenhaus stellen für die Betroffenen in der Regel eine erhebliche psychische und physische Belastung dar [22–24] und sollten nur bei ausreichender Indikation für eine stationäre Behandlung stattfinden. Die 5 häufigsten Behandlungsanlässe (laut Entlassdiagnosen) von Pflegeheimbewohnenden im Krankenhaus gemäß den auf GKV-Routinedaten basierenden Analysen des Projekts *Bedarfsgerechte Versorgung von Pflegeheimbewohnern durch Reduktion Pflegeheim-sensitiver Krankenhausfälle (PSK) *sind Herzinsuffizienz, Pneumonien, Femurfrakturen, Volumenmangel sowie sonstige Erkrankungen des Harnsystems. Sie waren bei einem Fünftel (20,5 %) aller Krankenhausaufenthalte (*n* = 191.174 Fälle) der Studienpopulation dokumentiert [25]. Ergebnis des PSK-Projekts ist schließlich eine Listung von 15 Erkrankungen (58 Diagnosen) zur Identifizierung potenziell vermeidbarer Hospitalisierungen von Pflegeheimbewohnenden – von jenen Leistungen also, die mittels einer optimierten Behandlung im Setting Pflegeheim, insbesondere durch ein wirksames berufsgruppenübergreifendes Zusammenarbeiten, vermeidbar wären. Diese Liste reicht von Diabetes mellitus Typ 2 über Niereninsuffizienz und Demenz bis hin zu Rückenschmerzen und oberflächlichen Verletzungen [25]. Fassmer und Pulst berichten Ähnliches aus dem Projekt *Hospitalisierung und Notaufnahmenbesuche von Pflegeheimbewohnern* (HOMERN; [26]); hier wird die jährliche Inzidenz von ungeplanten Krankenhausaufenthalten mit 1,7 pro Bewohnende (*n* = 1665) angegeben – bei je einem Drittel der Fälle lagen ein verschlechterter Allgemeinzustand (35 %) bzw. Stürze, Unfälle oder Verletzungen (34 %) zugrunde. Die Autorin und der Autor verweisen zudem auf die Relevanz von Patientenverfügungen, um ungeplante Krankenhausaufenthalte zu verringern [26].

## Zusammenfassung und Fazit

Der vorliegende Beitrag beleuchtet die Pflegebedürftigkeit in Deutschland sowie die Inanspruchnahme pflegerischer und gesundheitlicher Leistungen. Rund 6,3 % der gesetzlich Versicherten galten Ende des Jahres 2021 als pflegebedürftig. Im Jahr 2012 lag dieser Anteil noch bei 3,5 %. Die Demografie begründet den Anstieg gleichwohl nicht allein. Altersbereinigt und ohne die auf dem neuen niedrigschwelligen Pflegegrad 1 beruhenden Entwicklungen verbleibt ein Anstieg der Pflegebedürftigkeit um 40 %. Die Inanspruchnahme von Leistungen ist derweil relativ konstant geblieben. Der Anteil vollstationär Pflegebedürftiger ist aufgrund der überproportionalen Zunahme ambulant Versorgter seit 2015 von 26 % auf 21 % gesunken, absolut jedoch nur leicht zurückgegangen. Bei den ambulanten Unterstützungs- und Entlastungsleistungen ist zuvorderst die Nutzung der stundenweisen Verhinderungspflege und des Entlastungsbetrags gestiegen. Ferner nutzen weiterhin knapp 2 Drittel der Pflegegeldbeziehenden bzw. 40 % aller Pflegebedürftigen keine einzige weitere ambulante Unterstützungs- und Entlastungsleistung.

Die hier dargelegte Beschreibung von Pflegebedürftigkeit und -inanspruchnahme auf Basis von amtlichen Statistiken und Abrechnungsdaten der gesetzlichen Pflege- und Krankenkassen verweist gleichwohl auf bestehende Limitationen: Unser Verständnis von Langzeitpflegebedürftigkeit ist stark geprägt durch dessen sozialrechtliche Normierung. Die Berichterstattung auf Basis dieser Daten ist insofern auch geprägt von der Reform dieser Normierung – der Einführung des gesetzlich neuen Pflegebedürftigkeitsbegriffs im Jahr 2017. Vor dem Hintergrund der präsentierten Erkenntnisse wird somit der Forschungsbedarf deutlich: Die epidemiologische, primärdatenbasierte, bevölkerungsbezogene Erhebung von Pflegebedürftigkeit sowie die Erforschung der Bedingungen, welche die Inzidenz und Prävalenz von Pflegebedürftigkeit und auch deren Schwere (über die Zeit) beeinflussen. Das Spektrum möglicher Prädiktoren reicht von angebots- und nachfrageinduzierten über sozioökonomische bis hin zu gesellschaftlich-normativen. Zu verweisen ist hier auf das Potenzial von Primärdaten und -studien, die auf Daten des Deutschen Alterssurveys (DEAS) oder auch auf Pflegeeinstufungsinformationen des Medizinischen Dienstes beruhen, oder die aktuelle Hochaltrigenstudie D80+ als bevölkerungsrepräsentative Befragung, bei der Personen in Privathaushalten und Institutionen ab 80 Jahren interviewt wurden [27, 28]. Um den Herausforderungen aufgrund von steigendem Pflegebedarf in den nächsten Jahrzehnten begegnen zu können, ist ein tieferes Verständnis von Pflegewahrscheinlichkeiten und -ursachen unumgänglich.

Deutlich wird zudem: Pflegebedürftige betagte Menschen sind eine immanente Gruppe von Nutzenden unseres Gesundheitssystems. Ihre Zahl und der daraus abgeleitete komplexe Bedarf an vernetzter, interdisziplinärer und intersektoraler Versorgung sind eine der zentralen Herausforderungen in unserer alternden Gesellschaft. Die Diskontinuitäten an den Schnittstellen der gesundheitlichen Versorgung stehen zunehmend im Fokus. Während nahezu jede pflegebedürftige Person mindestens einen vertragsärztlichen Kontakt pro Quartal aufweist, verbleiben rund 28 % der Pflegebedürftigen ohne fachärztlichen Kontakt. Kritische Befunde, die den Bedarf einer Versorgungsoptimierung anzeigen, finden sich ebenso an der Schnittstelle der Arzneimittelversorgung: 15 % der Pflegebedürftigen erhielten PRISCUS-Wirkstoffe, 17 % Antipsychotika. Ferner markieren Krankenhausaufenthalte eine von Pflegebedürftigen häufig frequentierte Schnittstelle der Versorgung: Im Jahr 2020 gingen 44 % aller Krankenhaustage auf die Behandlung Pflegebedürftiger zurück. Zu Hospitalisierungen und Notfalleinweisungen von Gepflegten listet allein der Innovationsausschuss des Gemeinsamen Bundesausschusses (G-BA) zahlreiche geförderte Projekte auf. Auch dem Thema End-of-Life-Care wird – nimmt man wiederum die Förderung durch den Innovationsfonds als Maßstab – zunehmend mehr Beachtung geschenkt. Fragen mit Blick auf die Balance von kurativen Perspektiven und palliativen Ansprüchen an (Für‑)Sorgen, Pflegen und Lindern – unter Beachtung der Versorgungswünsche der Betroffenen und Angehörigen – bedürfen dennoch weiterhin intensiver Forschung.

## Supplementary Information





## Footnotes

1 Während die PRISCUS-Liste kürzlich in überarbeiteter Form als PRISCUS 2.0 erschienen ist, befindet sich diese Leitlinien gegenwärtig weiterhin im Status der Aktualisierung.
