# Supplementary material for: Epidemiology of long-term care: prevalence and utilisation as well as the healthcare of long-term care recipients in Germany
Source: Bundesgesundheitsblatt Gesundheitsforschung Gesundheitsschutz. 2023 Apr 27;66(5):479–89. [Article in German] doi: 10.1007/s00103-023-03693-5 (PMC10136379; doi:10.1007/s00103-023-03693-5)
Supplement: Supplementary file 1 [file 103_2023_3693_MOESM1_ESM.docx]

# Epidemiologie der Pflege: Prävalenz und Inanspruchnahme sowie die gesundheitliche Versorgung von Pflegebedürftigen in Deutschland

Abbildung Z1: Anteil Pflegebedürftiger (in Prozent) in der Gesetzlichen Krankenversicherung (GKV) nach Alter und Geschlecht im Zeitraum 2017-2021 (inkl. Pflegebedürftige, die Pflege in vollstationären Einrichtungen der Hilfe für behinderte Menschen nach § 43a Elftes Buch Sozialgesetzbuch [SGB XI] erhalten).

*Quelle: Amtliche Statistik PG2, Amtliche Statistik KM6*

Abbildung Z2: Anteil der Pflegebedürftigen (in Prozent) an den gesetzlich Versicherten nach Altersgruppen und Geschlecht, 2021 (inkl. Pflegebedürftige, die Pflege in vollstationären Einrichtungen der Hilfe für behinderte Menschen nach § 43a Elftes Buch Sozialgesetzbuch [SGB XI] erhalten).

*Quelle: Amtliche Statistik PG2, Amtliche Statistik KM6*

Abbildung Z3: Anteil der Pflegebedürftigen (in Prozent) nach Schwere der Pflegebedürftigkeit im Zeitraum 2017-2021 ((inkl. Pflegebedürftige, die Pflege in vollstationären Einrichtungen der Hilfe für behinderte Menschen nach § 43a Elftes Buch Sozialgesetzbuch [SGB XI] erhalten).

*Quelle: Amtliche Statistik PG2, Amtliche Statistik KM6*
